# *Babesia microti* from humans and ticks hold a genomic signature of strong population structure in the United States

**DOI:** 10.1186/s12864-016-3225-x

**Published:** 2016-11-07

**Authors:** Giovanna Carpi, Katharine S. Walter, Choukri Ben Mamoun, Peter J. Krause, Andrew Kitchen, Timothy J. Lepore, Ankit Dwivedi, Emmanuel Cornillot, Adalgisa Caccone, Maria A. Diuk-Wasser

**Affiliations:** 1 Department of Epidemiology of Microbial Diseases, Yale School of Public Health, New Haven, CT 06520 USA; 2 Department of Ecology and Evolutionary Biology, Yale University, New Haven, CT 06520 USA; 3 Department of Molecular Microbiology and Immunology, Johns Hopkins Bloomberg School of Public Health, Baltimore, MD 21205 USA; 4 Department of Internal Medicine, Section of Infectious Diseases, Yale School of Medicine, New Haven, CT 06520 USA; 5 Department of Anthropology, University of Iowa, Iowa City, IA 52242 USA; 6 Nantucket Cottage Hospital, Nantucket, MA 02554 USA; 7 Institut de Biologie Computationnelle, University de Montpellier, 34095 Montpellier, Cedex 5 France; 8 Department of Ecology, Evolution and Environmental Biology, Columbia University, New York, NY 10027 USA

**Keywords:** Apicomplexan, Tick-borne pathogen, Hybrid capture, Population genomics, Coalescent analysis

## Abstract

**Background:**

*Babesia microti* is an emerging tick-borne apicomplexan parasite with increasing geographic range and incidence in the United States. The rapid expansion of *B. microti* into its current distribution in the northeastern USA has been due to the range expansion of the tick vector, *Ixodes scapularis*, upon which the causative agent is dependent for transmission to humans.

**Results:**

To reconstruct the history of *B. microti* in the continental USA and clarify the evolutionary origin of human strains, we used multiplexed hybrid capture of 25 *B. microti* isolates obtained from *I. scapularis* and human blood. Despite low genomic variation compared with other Apicomplexa, *B. microti* was strongly structured into three highly differentiated genetic clusters in the northeastern USA. Bayesian analyses of the apicoplast genomes suggest that the origin of the current diversity of *B. microti* in northeastern USA dates back 46 thousand years with a signature of recent population expansion in the last 1000 years. Human-derived samples belonged to two rarely intermixing clusters, raising the possibility of highly divergent infectious phenotypes in humans.

**Conclusions:**

Our results validate the multiplexed hybrid capture strategy for characterizing genome-wide diversity and relatedness of *B. microti* from ticks and humans. We find strong population structure in *B. microti* samples from the Northeast indicating potential barriers to gene flow.

**Electronic supplementary material:**

The online version of this article (doi:10.1186/s12864-016-3225-x) contains supplementary material, which is available to authorized users.

## Background

*Babesia microti*, a tick-borne apicomplexan parasite, is the primary causative agent of human babesiosis, an emerging malaria-like illness in the northeastern and upper midwestern United States [1, 2]. Human babesiosis presents with a wide array of clinical severity, from asymptomatic infection in about a quarter of adults to fatal disease in up to 21 % of immunocompromised individuals [1, 3, 4]. *Babesia microti* is the most common pathogen transmitted through blood transfusion in the U.S. [5–7]. The first human case of babesiosis in an immunocompetent person was identified in 1969 on Nantucket Island, Massachusetts. The causative species was *B. microti*, which is now recognized as the most common *Babesia* spp. causing disease in humans [1].

In the United States, the enzootic transmission cycle of *B. microti* is similar to that of the etiologic agent of Lyme disease (*Borrelia burgdorferi*) and includes the blacklegged tick vector (*Ixodes scapularis*) and a range of vertebrate reservoir hosts, primarily the white-footed mouse (*Peromyscus leucopus*) [8]. The current invasion of *B. microti* and *B. burgdorferi* in the Northeast has followed the spread of their shared vector, *I. scapularis*, out of coastal refugia in Massachusetts and Rhode Island [9, 10]. Epidemiological modeling studies have estimated that although these diseases are spreading at a similar rate, Lyme disease dissemination had preceded that of babesiosis, which may reflect differences in transmissibility, differing reservoir host competences or differential barriers to spread [11].

Evaluation of genetic variation and structure of *B. microti* across its endemic range will shed light on the evolution, spread and transmission dynamics of this pathogen. Prior attempts to describe genetic diversity and relatedness of *B. microti* strains have been hampered by the conserved nature of the genetic markers used; both single locus (18S ribosomal DNA, rDNA, β-tubulin) and variable nucleotide tandem repeats (VNTR) [12–14]. These loci provide limited resolution at the spatio-temporal scale necessary for describing the relatively recent *B. microti* invasion in northeastern U.S. because of their low genetic diversity. Whole genome level analyses provide a powerful tool to explain origin, patterns and dynamics of spread, as they contain more genetic diversity than individual genes [15]. The first complete genome sequence of a *B. microti* isolate was reported in 2012 [16] and showed that the parasite is significantly distant from other apicomplexan taxa, including *Babesia bovis* and *Theileria* species. The genome of *B. microti* consists of 4 chromosomes, 1 linear mitochondrial genome and 1 circular apicoplast. The nuclear genome is ~6.5 megabases (Mb), the smallest apicomplexan genome sequenced to date, while the mitochondrial and apicoplast genomes are 11.1 and 28.7 Kb long, respectively [16–18]. A recent estimate of *B. microti* genome diversity was made using clinical samples [19], however no study has estimated *B. microti* diversity from natural populations of both human hosts and tick vectors.

Here, we describe genome-wide diversity, population structure and phylogenetic relationships of *B. microti* strains obtained from both individual field-collected ticks and human blood samples in babesiosis endemic and emerging areas in the Northeast and upper Midwest. We applied a hybrid capture assay to enrich for near-full-length *B. microti* genomes and co-capture *B. burgdorferi* from naturally infected vector and human populations [20]. Using this approach, we were able to survey pathogen genomic diversity directly from mixed DNA samples of tick vectors or human blood, precluding propagation in culture or immunodeficient rodents, methods known to introduce strain biases. Furthermore, by harnessing available genomic data we were able to assess for the first time the full spectrum of genomic diversity of co-infecting pathogens [21]. Genome-wide analyses of the newly sequenced *B. microti* genomes from the continental U.S. demonstrate that *B. microti* is structured into subpopulation clusters and provides new insights into the evolutionary history and origin of *B. microti* strains.

## Results

### Whole genome capture of tick- and human-derived B. microti*B. microti* strains

**Fig. 1:**
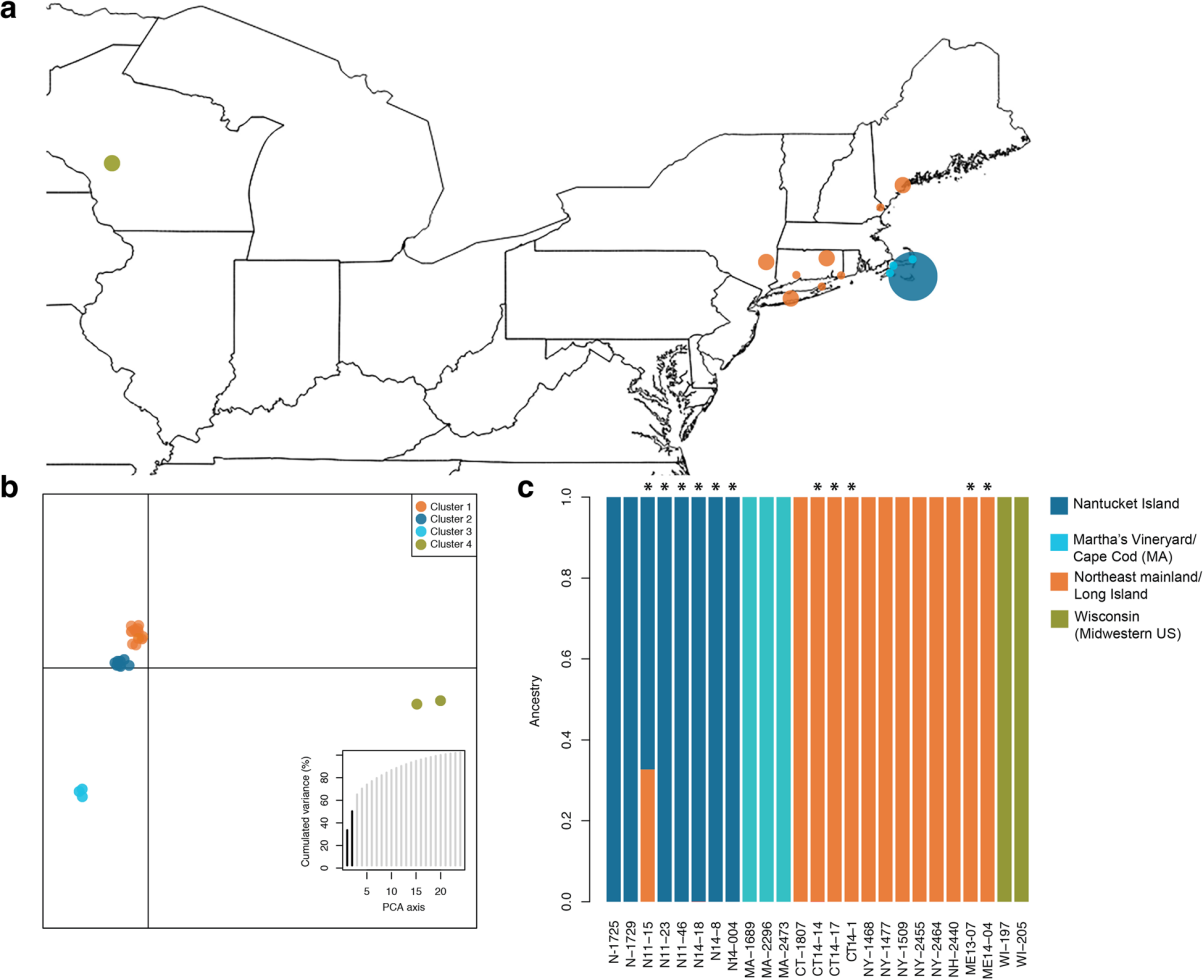
Population structure of *B. microti* in the continental U.S. **a** Map showing the geographic origin and the proportions of sample belonging to each cluster for each sampling site in the continental U.S. as determined by the DAPC analysis. Area of the circle is proportional to the sample size for the site. **b** Scatter plot showing the first two discriminant functions of the discriminant analysis of principal components applied to the *B. microti* genome-wide SNPs data set from the 25 samples (K = 4). Circles represent individual samples. *B. microti* samples originated from Nantucket Island (cluster 1) are indicated in dark blue, mainland Northeast sites (CT) Maine (ME), Long Island (NY) and New Hampshire (NH) (cluster 2) in orange, southeastern portion of Massachusetts, specifically Marta’s Vineyard Island and Cape Cod (MA) (cluster 2) in light blue, and the upper Midwestern samples (Wisconsin) (cluster 4) in green. The histogram shows the two principal components of PCA (x-axis) which contained 58 % of the data variance (y-axis) using K =4 as a prior clustering. **c**. Bar plots showing for each sample ancestral probability using ADMIXTURE on the genome-wide SNP dataset (K = 4) (14 tick-derived strains and 11 clinical isolates from human patients marked with “*”)

We performed whole genome capture for 44 *B. microti* strains from 33 field-collected infected nymphal ticks and 11 human samples. *B. microti* load, measured by qPCR, was highly variable in field-collected nymphal tick samples (median = 2,314 genome equivalents; range = 14.7–22,259) and in humans samples (median: 71,551 genome equivalents range: 2344–737,340) (Additional file 1: Table S1). The mean capture efficiency for the 44 samples (the proportion of sequence reads mapping to the *B. microti* R1 reference genome) was 51.6 % (0.31–98.4 %) with a mean genome coverage of 161X (range: 0.10–1,161X) (Table 1). *B. microti* load in starting samples (prior to hybrid capture) was a significant predictor of *B. microti* genome coverage in a quasi-Poisson model (*p* < 0.001) (Additional file 1: Figure S1). Coinfection with *B. burgdorferi* in ticks was a significant negative predictor for *B. microti* capture efficiency (*p* = 0.0002) and average genome coverage (*p* = 0.0292) in a quasi-Poisson model. For analysis of polymorphisms, we included samples with at least 72.8 % coverage of the genome (4.7 Mb of the 6.4 Mb reference genome) at minimum read depth of 10X, excluding telomeric regions, resulting in 25 *B. microti* isolates from 12 sites in northeastern U.S. and one site in the upper Midwest (Table 1, Fig. 1a). The 25 *B. microti* genomes (14 tick- and 11 human-derived strains) had a mean genome coverage of 366X (range: 14–1,937X).

**Table 1:** Whole-genome capture of *B. microti* from mixed DNA templates of tick and human samples

Sample	Site	State	Collection year	Source	*B. microti* genome copies^a^	Capture efficiency	Reference covered (%)	Mean Chr coverage	Mean apicoplast coverage
CT14-14	Mansfield	CT	2014	human blood	685,844	98.45	98.9	1937.3	1322.2
N11-23	Nantucket	MA	2011	human blood	164,819	97.75	98.8	1808.0	2168.8
N11-46	Nantucket	MA	2011	human blood	81,207	97.49	98.8	1402.1	1291.3
ME14-04	Portland	ME	2014	human blood	389,507	97.15	98.8	1071.0	714.8
ME13-07	Portland	ME	2013	human blood	136,878	96.14	98.8	480.0	202.0
N14-18	Nantucket	MA	2014	human blood	136,892	95.92	98.8	758.5	444.8
CT14-17	Mansfield	CT	2014	human blood	118,180	95.78	98.7	216.3	134.6
NY-1509	Mashomack Preserve	NY	2011	nymphal tick	22,259	95.53	98.7	176.5	598.8
CT-1807	Barn Island WMA	CT	2013	nymphal tick	NA	70.87	98.7	195.5	889.0
MA-2296	Quashnet River State Park	MA	2013	nymphal tick	9931	69.41	98.7	312.4	1003.2
N-1725	Squam Swamp	MA	2013	nymphal tick	6919	91.76	98.6	246.4	1135.4
N14-004	Nantucket	MA	2014	human blood	67,064	88.46	98.5	112.6	133.9
N14-8	Nantucket	MA	2014	human blood	1700	84.53	98.4	74.7	69.8
NY-1477	Connetquot State Park	NY	2011	nymphal tick	NA	80.66	98.4	63.6	105.1
WI-205	Black River State Forest	WI	2011	nymphal tick	NA	45.91	97.9	43.8	107.7
N-1729	Squam Swamp	MA	2013	nymphal tick	NA	48.11	97.0	33.8	173.1
NY-1468	Connetquot State Park	NY	2011	nymphal tick	2314	16.26	96.4	26.5	69.0
NY-2455	James Baird State Park	NY	2011	nymphal tick	NA	66.68	95.8	26.4	58.1
MA-1689	Manuel F. Correllus State Forest	MA	2013	nymphal tick	2256	86.12	94.8	34.3	248.6
MA-2473	Nickerson State Park	MA	2013	nymphal tick	4904	84.35	93.7	22.5	23.6
NH-2440	Great Bay National Wildlife Refuge	NH	2011	nymphal tick	7454	58.49	93.6	35.4	67.3
NY-2464	James Baird State Park	NY	2011	nymphal tick	NA	43.7	92.3	20.5	44.0
N11-15	Nantucket	MA	2011	human blood	2218	65.07	90.0	18.9	12.8
CT14-1	Woodbridge	CT	2014	human blood	8,025	85.84	81.9	15.4	11.0
WI-197	Black River State Forest	WI	2011	nymphal tick	NA	46.74	74.0	14.2	7.9
NY-2539	Hither Hills State Park	NY	2013	nymphal tick	NA	3.25	8.6	33.0	23.2
NY-2458	James Baird State Park	NY	2013	nymphal tick	3191	81.95	4.5	4.2	4.3
MA-1670	Manuel F. Correllus State Forest	MA	2013	nymphal tick	NA	20.06	2.2	1.2	63.5
CT-2493	Rocky Neck SP	CT	2013	nymphal tick	NA	5.97	2.1	0.6	8.0
NY-2387	Ward Pound Ridge	NY	2013	nymphal tick	268	17.22	1.9	0.4	7.0
NY-1524	Montauk Point State Park	NY	2013	nymphal tick	59	8.48	1.9	0.4	9.3
RI-2584	Block Island	RI	2013	nymphal tick	NA	20.69	1.7	3.1	21.7
ME-2737	Cape Elizabeth	ME	2013	nymphal tick	324	10.63	1.6	0.3	6.0
MA-2678	Nickerson State Park	MA	2013	nymphal tick	NA	2.68	1.5	0.3	8.0
CT-1824	Barn Island WMA	CT	2013	nymphal tick	224	38.53	1.1	2.8	8.8
NJ-2719	Naval Weapons Station Earle	NJ	NA	nymphal tick	118	31.21	1.1	2.1	17.1
NY-1587	Mashomack Preserve	NY	2013	nymphal tick	NA	22.26	0.8	2.6	8.9
RI-2589	Robins Hollow	RI	2013	nymphal tick	105	5.99	0.6	0.2	9.2
CT-2413	50-Foot Cliff	CT	2013	nymphal tick	200	14.05	0.4	0.0	2.0
WI-A406	Jackson County	WI	NA	female adult tick	NA	39.53	0.4	0.6	0.4
CT-2498	Rocky Neck SP	CT	2013	nymphal tick	NA	19.22	0.2	0.0	0.4
NY-2600	Hither Hills State Park	NY	2013	nymphal tick	15	0.31	0.2	0.3	6.4
WI-A118	Spooner Veterinary Clinic, Washburn Co.	WI	NA	female adult tick	NA	3.74	0.1	0.2	2.0
WI-A424	Jackson County	WI	NA	female adult tick	NA	15.44	0.1	0.0	0.2

Sample name, sampling site, state, collection year, source (nymphal or female adult *I. scapularis* tick or human blood), q-qPCR determined *B. microti* genome copy number, mean capture efficiency (the proportion of sequence reads mapping to *B. microti* R1 reference genome, GCF_000691945.1) percentage of genome covered (genome size 6.4 Mb), mean chromosomal coverage and mean apicoplast coverage (genome size 28.7 Kb). Samples are ranked by percentage of reference genome covered. The first 25 *B. microti* samples with at least 74 % coverage of the reference genome at minimum read depth of 10X were further used for downstream analyses

^a^For each sample, q-PCR determined *B. microti* genome copy number are shown for two techinical replicates

### Genomic diversity

Using SNP calling criteria described in the methods section, we detected 3,767 variants (SNPs, Single Nucleotide Polymorphisms and Indels) at the chromosomal level for this data set. Additional file 1: Figure S2 shows the distribution of the nucleotide diversity (π) per sliding window of 10-kb regions along the four chromosomes of *B. microti* for the 25 sequenced genomes. On average among the four chromosomes, we found a SNP density of 5.9 SNPs per 10,000 bp.

The complete apicoplast genome sequences (genome size: 28.7 Kb) had a mean genome coverage of 441X (range: 8–2,169X) (Table 1) with a range of 11–144 SNPs/apicoplast genome. The mean pairwise differences per site (π), among the 25 *B. microti* apicolplast genomes was π = 9.4 × 10^−4^ (SD = 3.9 × 10^−4^) (Table 2). We identified 17 unique apicoplast haplotypes composed of 167 segregating sites. The mean haplotype diversity for the apicoplast genomes was *Hd* = 0.943 (SD = 0.031, Table 2). Nucleotide and haplotype diversity did not significantly differ between the three clades identified by the Bayesian phylogenetic and network analyses (see below), a result that may have been due in part to the difference in sample sizes (Additional file 1: Table S2).

**Table 2:** Summary statistics for polymorphism in apicoplast genomes (28.7 bp)

Statistic	All	Wisconsin	Martha’s Vineyard/Cape Cod	Northeast
No. Samples	25	2	3	20
No. polymorphic (segregating) sites, *S*	167	42	4	21
No. haplotypes, *h*	17	2	3	12
Haplotype (gene) diversity, *Hd*	0.943	1	1	0.911
Std. dev, haplotype diversity	0.031	0.5	0.272	0.045
Nucleotide diversity, Pi	1.14E-03	1.47E-03	9.00E-05	1.60E-04
Std. dev, Pi	3.90E-04	7.30E-04	3.00E-05	2.00E-05
Watterson’s theta (per site)	1.55E-03	1.47E-03	8.00E-05	2.10E-04
Std. dev, Watterson’s theta	5.00E-04	1.10E-06	7.00E-05	8.00E-05
Fu & Li’s *D*	0.014 §	-	-	−1.219 §
Fu & Li’s *F*	−0.582 §	-	-	−1.279 §
Tajima’s *D*	−1.57 §	-	-	−1.567 §

**Fig. 2:**
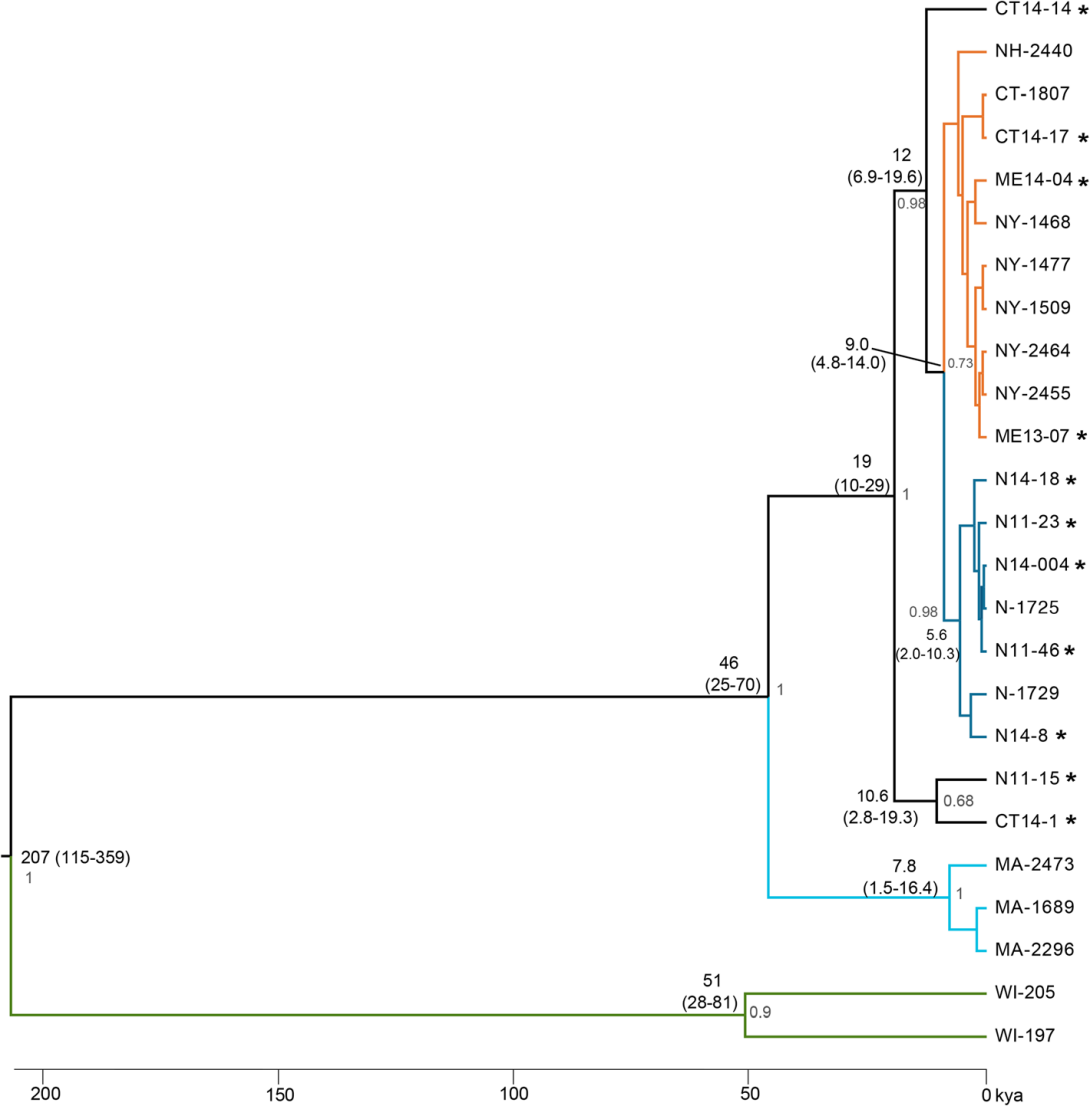
Phylogenetic reconstruction of 25 *B. microti* samples. Bayesian maximum clade credibility phylogeny of complete apicoplast genome sequences (28.7 Kb) from the 25 *B microti* samples calculated from the posterior distribution of trees generated by Bayesian MCMC coalescent analysis in BEAST [69]. Bayesian coalescent analysis was performed with a substitution rate of 1.2 × 10^−8^ substitutions per site per year (1.2 % per million years). Branches defining major clades are displayed in different colors, which correspond to the colors in Fig. 1 (b, c). Divergence dates (median estimates and 95 % HPD) are given in parenthesis for major nodes. Posterior probabilities > 0.65 are indicated at each node. The timescale is indicated below the phylogeny. *Babesia microti*-human derived samples are marked with “*”

Measures of diversity for all sampled *Babesia microti* and the three clades identified in the Bayesian phylogeny (Fig. 2). Statistics require sample size > 4 and are therefore not determined for populations from Midwest and Martha’s Vineyard/Cape Cod

§ Fu & Li’s *D,* Fu & Li’s *F* and Tajima’s *D* test statistics are non-significant (*p*-values > 0.10)

### Population structure and differentiation

The genome-wide SNP analyses showed strong genetic differentiation among geographic isolates, despite the relatively low genome-wide diversity across the sequenced *B. microti* samples. Discriminant analysis of principal components (DAPC) conducted on the genome-wide SNPs from the 25 *B. microti* samples revealed both continental and population specific patterns of genetic variation. Four main clusters were identified with the two principal components retained accounting for 58 % of the variance (Fig. 1a, b, Additional file 1: Figures S3 and S4). The first discriminant function distinguishes the Northeast from the Wisconsin *B. microti* samples. The second discriminant function differentiates the Northeast mainland (New England and NY state) and Long Island (hereafter ‘Northeast’) and the Nantucket Island from those from Martha’s Vineyard Island/Cape Cod samples in southeastern Massachusetts (MA, in Fig. 1b). The largest two clusters include both tick- and human-derived samples from the Northeast (mainland New England, New York and Long Island) (cluster 1, *n* = 12) and Nantucket Island (cluster 2, *n* = 8). The third cluster comprises tick-derived *B. microti* samples from Martha’s Vineyard Island/Cape Cod (MA) (cluster 3, *n* = 3). Cluster four encompasses the two tick-derived samples from Wisconsin (Fig. 1a, b).

To estimate population structure, probability of ancestry and admixture levels in each *B. microti* sample, we conducted model-based population structure analysis implemented in the ADMIXTURE program [22]. This analysis identified the same clusters found in the DAPC analysis (Additional file 1: Figure S5, Fig. 1c)*.* As in the DAPC analysis, human-derived samples grouped into two distinct clusters and are genetically more similar to non-human isolates than to each other, suggesting that human infectivity is not constrained to a single *B. microti* clade. The majority of samples did not show sign of genetic admixture with the exception of a human sample from Nantucket Island (N11-15; Fig. 1c).

*F*_*ST*_ estimates between clusters 1 (Northeast) and 2 (Nantucket Island), which include both clinical and tick-derived isolates, were low (*F*_*ST*_ = 0.136) and associated with a modest number (*n* = 95) of diagnostic (fixed in alternative states) SNPs. Similar estimates for comparison between clusters 2 (Nantucket Island) and 3 (Martha’s Vineyard Island/Cape Cod) and clusters 1 (Northeast) and 3 (Marta’s Vineyard Island/Cape Cod) revealed high levels of differentiation (Cluster 2–3: *F*_*ST*_ = 0.456, 504 diagnostic SNPs; cluster 1–3: *F*_*ST*_ = 0.415 and 519 diagnostic SNPs) (Additional file 1: Table S3).

### Phylogenetic analysis and divergence time estimation

To investigate the phylogenetic relationships among the 25 *B. microti* samples, we focused on the apicoplast genome (28.7 Kb) due to the presence of few repetitive elements and a coding density of over 98 % [18]. We constructed a maximum likelihood (ML) tree and conducted a Bayesian coalescent analysis on the 25 complete apicoplast genomes using the mutation rate estimated for the most closely related apicomplexan parasite available, *Plasmodium falciparum:* 1.2 × 10^−8^ substitutions/site/year (1.2 % per million years;[23]). The ML tree identified three distinctive clades (Additional file 1: Figure S6). The Bayesian coalescent analysis produced a tree with concordant topology to the ML tree (Fig. 2). The most recent common ancestor between the Northeast and Wisconsin clades was estimated to have occurred 207,000 years ago (95 % Highest Posterior Density –HPD, 115–359). The most recent common ancestor of the Martha’s Vineyard Island/Cape Cod and the Northeast clades was estimated to have occurred 46,000 years ago (95 % HPD 25–70) (Fig. 2).

### Population demography

We constructed a TCS network [24] to assess the genealogical relationships between the circulating apicoplast haplotypes and to gain insight into the population level phenomena that might have contributed to the observed patterns (Additional file 1: Figure S7). This network confirms the topology obtained from the phylogenetic analyses (Additional file 1: Figure S7, Fig. 2). The statistical parsimony analysis did not connect all the 17 haplotypes into a single network (Additional file 1: Table S2). One of the three identified haplogroups (groups of related haplotypes), which includes the Wisconsin haplotypes, is separate and includes two divergent haplotypes (42 substitutions apart) and is not connected with the other two haplogroups. A second haplogroup includes all the haplotypes from Northeast except for the Martha’s Vineyard Island/Cape Cod haplotypes, which comprise the third haplogroup. The Northeast and Martha’s Vineyard Island/Cape Cod haplogroups are genetically distinct from each other (20 substitutions apart). While the Northeast haplogroup encompassed several divergent haplotypes (from 1 to 19 substitutions), the three haplotypes within the Martha’s Vineyard Island/Cape Cod haplogroup differ by up to 5 substitutions. However, nucleotide diversity and haplotype does not significantly differ between the Northeast and Martha’s Vineyard Island/Cape Cod haplogroups (Additional file 1: Table S2).

The Bayesian Skyline Plot, which describes population changes as a function of time, suggests a *B. microti* population expansion within the past ~1000 years (Additional file 1: Figure S8, A and B). The strength of this inference might be limited because the Bayes Factors do not favor a Bayesian Skyline model over a constant population size model, possibly because of small sample size and low diversity of the samples.

We used several multiple population genetic statistics to test for historic changes in *B. microti* population size (Additional file 1: Tables S2 and S3). Tajima’s *D* and Fu & Li’s *F* statistics are expected to be 0 in neutrally evolving populations of constant population size and negative in populations undergoing purifying selection or demographic expansion. Tajima’s *D* and Fu & Li’s *F* statistics on apicoplast haplotypes were negative (though not significantly different from 0) for all *B. microti* samples, as well samples from the Northeast clade. These findings potentially indicate historic population expansion but sample sizes were too small for accurate measurement in the Wisconsin and Martha’s Vineyard Island/Cape Cod clades. The mismatch distribution is unimodal for all samples examined together (results not shown), suggesting demographic or spatial population expansion. We tested evidence for a spatial *B. microti* expansion by fitting a model of spatial expansion to the mismatch distribution in ARLEQUIN [25] and evaluated expansion model fit using the sum of square deviations (SSD) [26] and the raggedness index [27] (Additional file 1: Table S4). Here, non-significant values for SSD and for the raggedness index signify that the data do not deviate from that expected under the model of expansion. The hypothesis of spatial expansion of *B. microti* could not be rejected for all samples (SSD = 0.022, *P* = 0.80, Harpending’s raggedness = 0.025, *P* = 0.70) as well as in the Northeast clade (SSD = 0.11, *P* = 0.11, Harpending’s raggedness = 0.05, *P* = 0.62) (Additional file 1: Table S3). The analysis could not be carried out on the other clades because of their small sample size. The more distant estimated expansion time of all samples (*τ* = 137.44) compared to the Northeast samples (*τ* = 3.90) likely reflects ancient divergence between the Wisconsin and other samples and a more recent spatial expansion event in Northeast.

### Co-infection pattern with B. burgdorferi for human samples

We used a dual-pathogen genome capture array to enrich for multiple targeted parasite species within a single human or vector specimen to assess co-infections pattern with *B. burgdorferi.* Fifty percent of tick samples were known to be coinfected with *B. burgdorferi*. We had no a priori knowledge of the coinfection status of the human samples. Four out of the eleven human samples (~36 %; N14-004, N11-23, CT14-14, N11-16) had a mean *B. burgdorferi* chromosomal coverage of 7.8X (Additional file 1: Table S4) indicating that *B. burgdorferi* DNA was detectable in the blood of *B. microti* infected patients at the time of sampling.

## Discussion

Our sample of B. microti collected from a wide spatial range from both ticks and humans reveals higher levels of genetic diversity than previously reported using only single locus or microsatellite markers [12–14], and significantly lower diversity than other Apicomplexa parasites. This higher genetic resolution allowed us to identify fine-scale genomic differentiation and to examine the evolutionary and demographic history of an important emerging parasite.

### Comparison of *B. microti* genomic diversity with other apicomplexan taxa

Genome-wide diversity of *B. microti* is lower than that of other apicomplexan parasites. For comparison, SNP density observed in our data set (5.9 SNPs per 10,000 bp) is four-fold lower than estimates in other apicomplexan species. For example, in 16 geographically diverse *Plasmodium falciparum* parasites, a total of 46,937 SNPs (22.4 per 10,000 bp) were identified, demonstrating substantially higher genomic diversity compared to *B. microti* [28]. The low diversity of *B. microti* may be explained by non-mutually exclusive mechanisms as compared to *P. falciparum*, including the ecology of the transmission cycle and the demographic history of the parasite. *B. microti* is characterized by a low transmission rate compared to *P. falciparum*. The tick/mouse natural transmission cycle of *B. microti* spans a minimum of two years, with a single blood meal for pathogen acquisition as a tick larva and another blood meal for transmission as a tick nymph, typically about 11 months after the larval meal [29–31]. This low biting frequency reduces the likelihood of superinfection and thus opportunities for parasite recombination. In strong contrast, malaria mosquito vectors have a 3–4 day feeding cycle, which provide numerous opportunities for parasite infection and/or transmission and parasite diversity during the adult lifespan [32]. The other possible mechanism for the markedly low diversity in *B. microti* is a recent population bottleneck that occurred during the rapid conversion of forests to farmland and intensive hunting in New England from ~1800–1930, resulting in a well-documented reduction of white-tailed deer and tick populations [14, 33–36].

### Genetic differentiation among *B. microti* isolates

Despite low genome wide diversity, multivariate analyses of the genome-wide SNPs reveals strong population structure, with at least four major parasite genetic clusters in the USA, three of them in the Northeast (Fig. 1a and b). This significant geographic structure is consistent with the low dispersal capability of *B. microti*, given that the primary reservoirs are short-range migration hosts, such as rodents [11, 21]. Additionally, we found significant genomic divergence among *B. microti* populations within the Northeast. Samples from Northeast, Nantucket Island and Martha’s Vineyard Island/Cape Cod represented three distinct populations (Fig. 1c). Our findings on *B. microti* population structure in the continental US support the results of a recent study by Lemieux and colleagues [19]. We also found very limited evidence of genetic admixture among isolates from the northeastern clades (a single human sample from the Nantucket Island population; N11-15; Fig. 1c), consistent with a low dispersal capability of *B. microti* and thus a limited low gene flow between these populations.

### Phylogenetic analyses, timing of the divergence and demography

The ML and Bayesian phylogenetic analyses of the complete apicoplast genomes provide timing of divergence of the *B. microti* clades that were identified. On a continent-wide scale, our findings are consistent with the Northeast and Midwest *B. microti* clades having common ancestry in the Pleistocene, as long as ~200,000 years ago, and possibly more recently (Figs. 1a and 2), but cannot distinguish between either a west-to-east or east-to-west radiation of *B. microti* across North America. Inferring directionality of *B. microti* radiation requires inclusion of a more distantly related *Babesia* species to *B. microti* (i.e. *Babesia rodhaini* or *Babesia duncani*) as an outgroup, however their genomes have not been sequenced yet. At a regional scale, the ML and Bayesian phylogenetic analyses confirmed the genetic distinctiveness of the *B. microti* isolates from Martha’s Vineyard Island/Cape Cod compared to the isolates from Nantucket Island and from the Northeast sites, despite their geographic proximity (Fig. 2). These phylogenetic analyses also provided a time estimate for the divergence of the Martha’s Vineyard Island/Cape Cod and Nantucket Island clades. While the timing for the split between the Wisconsin and Northeastern samples dates back to ~ 207,000 years ago (95 % HPD x115-359), the split between Martha’s Vineyard Island/Cape Cod and Nantucket Island is relatively recent ~46,000 years (95 % HPD 25–70) (Fig. 2). Notably, we used an established mutation rate of 1.2 × 10^−8^ sub./site/year; [23]; and this mutation rate may be very conservative; however, there is currently a paucity of apicoplast substitution rate estimates. It is unclear which species of tick maintained *B. microti* over geologic timeframes. While *I. scapularis* is the dominant present day vector, other vectors may also have played a role, as there is evidence that *B. microti* was able to be maintained in the absence of *I. scapularis* in Martha’s Vineyard Island through a sylvatic cycle by the rodent-feeding nidicolous tick species, *Ixodes muris* or *I. angustus* [13, 14].

Sampled *B. microti* harbor a genomic signature of historic spatial expansion across all samples as well as for the Northeast clade (Additional file 1: Table S3), although our small sample size limits precise estimation of the timing of a historic population bottleneck (Additional file 1: Table S3). The Bayesian Skyline Plot suggests a *B. microti* population expansion in the northeastern U.S. that occurs within the past 1000 years. Although limited by sample size, the topology of the haplotype network (Additional file 1: Figure S7) suggests an older expansion of the Northeast clade than the one including the Martha’s Vineyard Island/Cape Cod haplogroups, which may suggest a west to east spread.

The combination of our genome-wide SNP approach, the coalescent based phylogenetic analyses of the apicoplast genomes, and the network analyses allowed us to describe patterns and timing of divergence among isolates found at both large and at fine spatial scale that were undetectable using previous methods [14]. Further sampling will likely yield a more refined understanding of invasion history, as well as increased monitoring of the levels of genetic admixture among strains. Such information may provide important epidemiological and clinical insights.

### The origin of human clinical isolates

Based on the genome-wide SNP analysis, clinical samples fall within two genetic clusters (Northeast and Nantucket Island), indicating that multiple *B. microti* variants circulating in tick populations are infectious to humans (Fig. 1b, c). Generating a genomic catalog of both human and tick-derived *B. microti* strains will provide a foundation for continued study of determinants of *B. microti* virulence in humans. Further genomic studies are needed to identify protein-coding loci or other genomic regions that could be more efficiently targeted for development of improved diagnostics, anti-parasitic compounds or identification of virulence factors.

### Multi-pathogen capture

Our study demonstrates that hybrid capture can simultaneously enrich and whole-genome sequence multiple targeted parasite species within a single human or vector specimen, allowing for study of the effect of multi-parasite epidemiological and of clinical outcomes. We found a pattern of coinfections with *B. burgdorferi* in ~36 % of the human samples. The observed coinfection rate is not unexpected given the high coinfection rate of *B. burdgorferi* in the northeastern tick populations (up to 18.6 % in adult *I. scapularis*), potentially driven by positive interactions between *B. microti* and *B. burgdorferi* in ticks and hosts [37]. Although our multiplexed hybrid capture method is highly sensitive and scalable, we determined that *B. microti* copy number in initial samples is a significant predictor for capture efficiency and genome coverage (Additional file 1: Figure S1), and we propose ~2300 *B. microti* genome equivalents as a conservative minimum parasite load estimate required in vector samples to achieve a genome coverage suitable for population genomics applications [38].

## Conclusions

We demonstrate that *B. microti* genomes reveal a signature of ancient evolutionary processes that place population-history divergence of the Northeast and Midwest *B. microti* populations at least back to the Pleistocene, while the northeastern clades diverged more recently. The association of human-derived samples to distinct, rarely intermixing clusters, raises the possibility that divergent infectious phenotypes affect humans, resulting in differential clinical outcomes and epidemiological patterns. Our work provides the foundation for more extensive sampling and genome wide association studies to explore the full implications of the observed genomic structure.

## Methods

### Sample collection

*B. microti*-infected ticks were sampled from a large spatial range reflecting the current geographical distribution of *B. microti* in the United States and hypothesized ancestral sites of its tick vector [33]. Samples include 33 *B. microti* strains derived from host-seeking *I. scapularis* nymphs from 25 sites from the continental U.S. (New York, Connecticut, Massachusetts, New Hampshire and Wisconsin) and 11 strains from peripheral blood samples obtained from patients from 3 sites in the northeastern U.S.: the original babesiosis focus, Nantucket Island and other currently endemic sites in Connecticut and Maine. Field collections of *I. scapularis*, DNA extraction from individual ticks and human blood samples, and qPCR testing for *B. microti* infection and parasite load determination were perfomred using previously described protocols [39, 40].

### Whole genome capture and sequencing

*B. microti* genomic DNA was enriched from mixed DNA templates of tick and human samples using a custom hybridization capture array following the NimbleGen SeqCap method (Madison, USA) [20]. Hybrid capture techniques overcome the need of propagation of *B. microti* in rodents or whole genome amplification, methods used to enrich pathogens prior to genome sequencing and known to introduce biases in strain representation and propagation of sequencing errors, respectively [41, 42]. We used a dual-genome pathogen capture array targeting *B. microti* and *B. burgdorferi* genomes. Custom DNA probes were designed *in silico* to tile 99.9 % of the *B. microti* R1 reference genome (GenBank: GCF_000691945.1; genome size 6.4 Mb, 4 chromosomes and 1 mitochodrion; GenBank: LK028575 apicoplast complete genome, 28.7 Kb) [16–18], while *B. burgdorferi*, probes were design as previously reported [20]. Illumina library preparation, hybridization capture and sequencing was conducted at the Yale Center for Genomic Analysis (YCGA). Briefly, library preparation for each sample was conducted using a modified Roche/Nimblegen SeqCap EZ Library Short Read protocol [43]. Library concentration was determined using PicoGreen assay (Invitrogen) and size selection was performed on a Caliper LabChip GX instrument (PerkinElmer). Equimolar amounts of each indexed genomic library were pooled in 10-plex prior to capture for a total of 1 ug total genomic DNA per hybridization reaction. Samples were heat-denatured and mixed with the custom DNA probes (Roche/NimbleGen) and hybridization performed at 47 °C for 68 h. Samples were then washed with a series of stringent buffers to remove non-specifically bound DNA fragments. The captured fragments were PCR amplified and purified with AMPure XP beads. Sample concentrations were normalized to 2 nM and loaded onto two lanes of Illumina version 3 flow cells at a concentration that yields 170–200 million passing filter clusters per lane. Samples were sequenced using 75 bp paired end sequencing on an Illumina HiSeq 2500 according to Illumina protocols. Sample de-multiplexing was performed using Illumina’ s CASAVA 1.8.2 software.

We used univariate logistic regression to detect correlates of *B. microti* capture efficiency and genome coverage. We used a quasi-poisson model to account for overdispersion. Statistical analyses were implemented in R version 3.1.1 (R Core. 2014).

### Read alignment and SNP detection

Illumina sequence reads for each sample were aligned to the newly annotated *B. microti* R1 reference genome and the apicoplast complete genome [18, 44] using BWA alignment tool [45] (v. 0.7.7, bwa mem, default parameters). PCR duplicates were identified and excluded from downstream analyses using Picard (v. 1.117) MarkDuplicates (http://picard.sourceforge.net/). Aligned reads were realigned around indels using GATK (v. 3.3) IndelRealigner [46, 47] and base quality scores of realigned reads were then recalibrated using GATK TableRecalibration. Genome coverage for each sample was calculated using GATK DepthOfCoverage (mmq > 20, mbp > 20). For downstream analyses we included samples for which >75 % of the *B. microti* R1 reference genome had at least 10X coverage, resulting in a total of 25 *B. microti* strains (13 tick- and 11 human-derived strains). Genetic variants (SNPs and INDELs) were identified using GATK UnifiedGenotyper and stringent filters were applied using GATK VariantFiltration to achieve a high confidence SNP set [(DP < 12) || (QUAL < 50) || (SB > −0.10) || (MQ0 > = 2 && (MQ0/(1.0 * DP)) > 0.1)]. SNPs identified in *B. microti* telomeres [16] were excluded to minimize potential false-positive variant calls due to low sequence complexity in these regions that precluded accurate short read mapping.

To determine co-infection pattern of human samples with *B. burgdorferi*, Illumina sequence reads for these samples were aligned to the *B. burgodorferi* B31 reference genome (910 Kb) and mapping statistics were calculated using the same methods and filtering parameters as described above.

### Population structure

Discriminant Analysis of Principal Components (DAPC), a non model-based method implemented in the adegent R package [48, 49] was used to describe genetic clusters using synthetic linear combination of variables (in this case SNP genotypes) that best differentiate between two or more groups of individuals. First, we ran sequential K-means clustering algorithm for K = 2 to K = 22 on the SNP data transformed by principal component analysis and subsequently clusters were identified using discriminant analysis (DA). We examined and compared the clustering solutions using Bayesian Information Criterion (BIC). Based on this analysis, K = 4 represented the optimal number of clusters to describe our *B. microti* SNP data set (Additional file 1: Figure S2). To minimize overfitting of the data we retained two principal components, representing 58.6 % of the total variance, following optimization procedure as recommended by the package developers [50] (Additional file 1: Figure S4).

We used ADMIXTURE [22] to estimate the optimum number of ancestral clusters in our dataset and to assess the ancestry of the 25 *B. microti* samples using all bi-allelic SNPs and a subset of SNPs thinned to remove those likely in LD [22]. Considering only fully called sites, *F*_*ST*_ was calculated in vcftools [51] using corrections for small sample size [52, 53].

### Apicolplast diversity and demographic history

The number of segregating sites, total number of mutations, number of haplotypes, haplotype gene diversity and two measures of nucleotide diversity, and *θ*_W_, [54, 55] were calculated using DnaSP v5 [56]. To establish neutrality and to examine demographic history of *B. microti*, Fu & Li’s *D* and *F* statistics [57] in addition to Tajima’s *D* [58] were calculated in DnaSP v5 [56]. In a population at demographic equilibrium, the mismatch distribution is expected to multimodal; in populations that recently passed through a demographic or spatial expansion, the mismatch distribution is expected to be unimodal [59, 60].

### Phylogenetic analysis and divergence time estimation

The 25 *B. microti* apicoplast genome sequences generated in this study were aligned using ClustalW [61] with default parameters, and the alignment was manually adjusted to maintain reading frame integrity in the protein coding genes. The resulting alignment contained 28,620 sites. The genealogical relationships among the apicoplast genomes were investigated using several approaches. First, we estimated phylogenetic networks using the statistical parsimony procedure implemented in the software TCS 1.2 [24] using the 95 % limit for a parsimonious connection. Phylogenetic analyses were conducted using maximum likelihood (ML) inference as implemented in MEGA v5.2.2 [62] and Bayesian coalescent analysis using the BEAST package v1.7.1 [63]. The program jModelTest was used to determine the most appropriate substitution models for both ML and Bayesian analyses (TIM + G + I was selected using the Bayesian information criterion). We conducted BEAST coalescent analysis to estimate the genetic divergence of our apicoplast genome sequences and determine whether our data reflect changes in *B. microti* population sizes. We used the known substitution rate for the most closely related apicoplexan parasite available to calibrate our phylogeny and date internal nodes. The used substitution rate derives from a mitochondrial substitution rate for *Plasmodium* of 1.2 × 10^−8^ sub./site/year (1.2 % per million years;[23]); a previous analysis of mitochondrial and apicoplast evolutionary rates suggested that they are broadly similar [64]. The application of this rate may be speculative; however, there is currently a paucity of apicoplast substitution rate estimates. All Markov chains were run for 20 million generations, sampled every 2000 generations and the first 10 % of each chain was discarded as burn-in period. Analyses were run in duplicate to ensure Markov chain convergence. Strict and relaxed clocks (uncorrelated lognormal clock [65] were used, as were constant and Bayesian Skyline Plot [66] demographic models. Default priors were used for most model parameters; the exceptions were broad lognormal priors applied to population sizes and truncated exponential priors on the variance of the uncorrelated lognormal clock. Clock and demographic models were compared using Bayes factors. Marginal likelihoods for all combinations of clock and demographic models were estimated using path sampling, stepping stone and harmonic estimators [67, 68]. The combination of a relaxed clock and a constant population size had the highest marginal likelihood, and received “strong” support over analyses with strict clock models; there was equivocal support for a constant over variable population history. All apicoplast phylogenetic results will reference the analysis with the TIM + G + I, uncorrelated lognormal clock and constant size models. The program Tracer v1.5 (http://tree.bio.ed.ac.uk/software/tracer/) was used to inspect the BEAST output for convergence.

To investigate the demographic history of *B. microti* samples, we tested for a signal of spatial expansion using the mismatch distribution as implemented in ARLEQUIN [25]. In a population at demographic equilibrium, the mismatch distribution is expected to multimodal; in populations that recently passed through a demographic or spatial expansion, the mismatch distribution is expected to be unimodal [59, 60].

## Additional file

Additional file 1: Supplementary Information. (DOCX 1525 kb)
