# Supplementary material for: Babesia microti from humans and ticks hold a genomic signature of strong population structure in the United States
Source: BMC Genomics. 2016 Nov 7;17:888. doi: 10.1186/s12864-016-3225-x (PMC5100190; doi:10.1186/s12864-016-3225-x)
Supplement: Additional file 1: — Supplementary Information. (DOCX 1525 kb) [file 12864_2016_3225_MOESM1_ESM.docx]

**Supplementary Information for**

***Babesia microti*from humans and ticks hold a genomic signature of strong population structure in the United States**

Giovanna Carpi ^1,2,3^, Katharine S. Walter ^1^, Choukri Ben Mamoun ^4^, Peter J. Krause ^1,4^, Andrew Kitchen ^5^, Tim Leopore ^6^, Ankit Dwivedi 7, Emmanuel Cornillot ^7^, Adalgisa Caccone ^1,2,§^, Maria

Diuk-Wasser ^1,8,^^§, §§^

^1^ Department of Epidemiology of Microbial Diseases, Yale School of Public Health, New Haven, CT 06520, USA

^2^ Department of Ecology and Evolutionary Biology, Yale University, New Haven, CT 06520, USA

^3^ Department of Molecular Microbiology and Immunology, Johns Hopkins Bloomberg School of Public Health, Baltimore, MD, 21205 USA

^4^ Department of Internal Medicine, Section of Infectious Diseases, Yale School of Medicine, New Haven, CT 06520, USA

^5^ Department of Anthropology, University of Iowa, Iowa City, IA, USA

^6^ Nantucket Cottage Hospital, Nantucket, Massachusetts, USA

^7^ Institut de Biologie Computationnelle, IBC, Université de Montpellier, 860 rue St Priest, Bat 5 - CC05019, 34095 Montpellier Cedex 5, France

^8^ Department of Ecology, Evolution and Environmental Biology, Columbia University, New York, NY 10027, USA

^§^  Contributed equally to the publication

^§§^ Corresponding author:

Maria Diuk-Wasser, address: Department of Ecology, Evolution and Environmental Biology, Columbia University, New York, NY 10027, USA

Email: [mad2256@columbia.edu](mailto:mad2256@columbia.edu)

**Supplementary Tables**

**Table S1**. Mapping statistics of *Babesia microti* from 44 mixed DNA templates of tick and human samples. For each sample the first six columns list the designations and geographic origins of each *B. microti* sample*,* source (nymphal *I. scapularis* tick or human blood), genomic DNA concentration, q-PCR determined *B. microti* genome copy number. The final three columns report the number of HiSeq2000 paired-end reads prior to filtering, number of PCR duplicates, and number of reads that mapped to the *B. microti* R1 reference genome, GCF_000691945.1).

**Table S2.** Estimated spatial expansion parameters for *Babesia microti.* Spatial expansion model parameters fit to the observed apicoplast mismatch distribution for all sampled *B. microti* and the three clades identified in the Bayesian phylogeny (Figure 2).

*Demographic expansion models did not converge for Wisconsin or Martha’s Vineyard Island/Cape Cod clades, likely due to small sample sizes.

**Table S3**. Population genetic statistics for the identified *Babesia microti* clusters in the continental U.S. *F_ST_* were calculated on the genome-wide SNP data set using corrections for small sample size (Weir and Cockerham, 1984).

**Table S4.** Mapping statistics of *B. burgdorferi* from the 11 *B. microti*-infected human samples using the dual-pathogen genome hybrid capture array. Four B. microti infected human samples (4/11; 36%) were found coinfected with *B. burgdorferi* given a genome coverage greater than 3X.

**Supplementary Figures**

**Figure S1.** *Babesia microti* genome coverage as a function of parasite load. Each point represents the number of *B. microti* genome equivalents log-transformed (measured by qPCR) and the y-axis represent the mean chromosomal coverage. *B. microti* source is indicated by color (human in red, *I. scapularis* nymphs in blue). *B. microti* copy number in the starting sample is a significant predictor of *B. microti* chromosomal coverage in quasi-Poisson model.

**
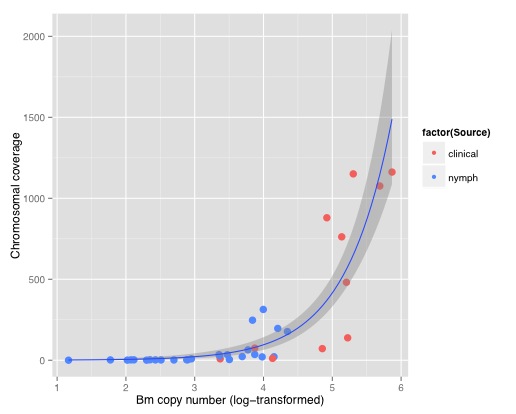
**

**Figure S2.** SNP density along the *Babesia microti* chromosomal genome. Distribution of the nucleotide diversity (pi) per sliding window of 10-kb regions along the four chromosomes of *B. microti* for the 25 sequenced genomes. The y-axis corresponds to nucleotide diversity per 10,000 bp window; the x-axis corresponds to the *B. microti* reference genome R1 chromosomal position; the red line indicates the average number nucleotide diversity.

**

**Figure S3**. Inference of the number of clusters in the DAPC performed on the *Babesia microti* SNP dataset. The graph shows the output of the function “find.clusters” used to identify the number of clusters in the *B. microti* SNP dataset. The Bayesian information criterion (BIC) is provided for different numbers of clusters, K, from 1 to 22. We considered K<22 because we are limited by sample size. A K value of 4 represents the best summary of the data.

**Figure S4.** Optimal number of PCs. α-score optimization values as a function of the number of retained PCs for the *Babesia microti* SNP dataset. The graph shows that only a few PCs (PCs=2) need to be retained for the assignment analysis.

**Figure S5.** Model-based ancestry analysis**.** ADMIXTURE analysis was performed to estimate the optimum number of clusters (k) in full dataset (3,767 SNPs). The graph illustrates the cross-validation errors for the number of ancestral populations k. Ancestry of each sample using k=4 clusters is reported in Figure 1C.

**Figure S6.** Maximum Likelihood phylogeny of the 25 *B. microti* samples. Phylogenetic relationships inferred from maximum likelihood analysis of the complete apicolpast sequences (28.6 Kb) (GTR+G+I). *Babesia microti-human derived samples are marked with “ * ”.*

**Figure S7.** Haplotype networks for the 25 apicoplast genomes obtained by TCS (Clement and Posada, 2000) showing the genealogical relationships based on a 95% parsimony criterion (parsimony limit =72) between the 17 apicoplast haplotypes found in this study. The statistical parsimony analysis did not connect all haplotypes into a single phylogenetic network. Instead, 2 main groups of haplotypes were observed, all the Northeast samples and the Wisconsin ones. Sampled haplotypes are shown as ovals or a rectangle (the inferred ancestral haplotype), with the name of the haplotype listed inside them and their size reflecting their frequency in the sample (Table S3). Inferred intermediate haplotypes that were not sampled are shown as small black circles, separated by segments, representing single substitution. Given the high number of substitutions between the haplotypes NH-2440 and MA-2296 (N=20) and the WI-205 and WI-197 (N=42) the connection between them was bracketed and not scaled for drawing convenience.

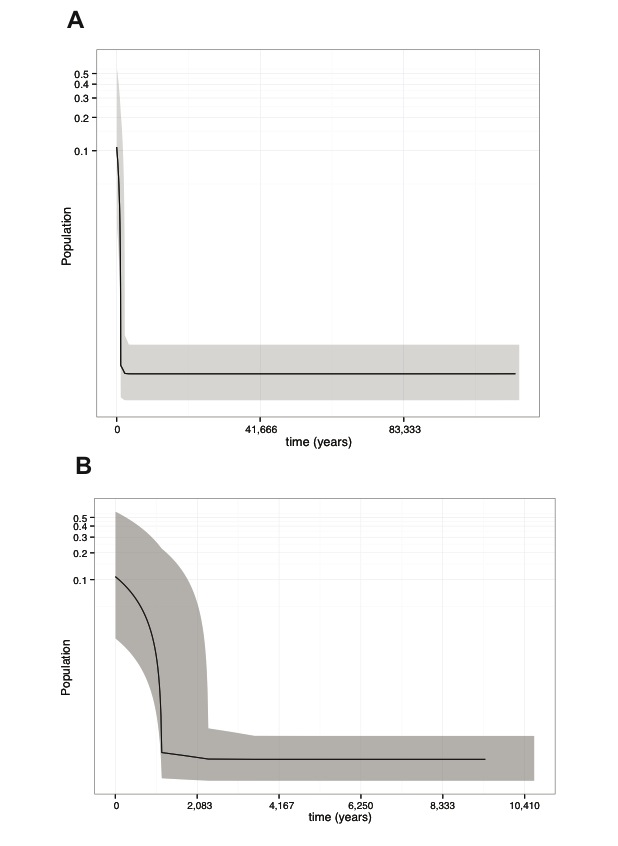
**Figure S8.** Bayesian Skyline Plot describing population changes as a function of time. (**A**) The y-axis represents the median population size (continuous line) and the 95% highest density probability (HDP) in shaded grey, and x-axis represent years backward in time from 0 to ~117,000 years. The y-axis is in logarithmic scale. (**B)** Bayesian Skyline Plot for the range of 0 to 10,410 years. The plot shows *B. microti* population expansion over the past ~1,000 years.
